# Differential Expression Patterns of *Pleurotus ostreatus* Catalase Genes during Developmental Stages and under Heat Stress

**DOI:** 10.3390/genes8110335

**Published:** 2017-11-21

**Authors:** Lining Wang, Xiangli Wu, Wei Gao, Mengran Zhao, Jinxia Zhang, Chenyang Huang

**Affiliations:** 1 Institute of Agricultural Resources and Regional Planning, Chinese Academy of Agricultural Sciences, Beijing 100081, China; wanglining90@126.com (L.W.); wuxiangli@caas.cn (X.W.); gaowei01@caas.cn (W.G.); zhaomengran@caas.cn (M.Z.); zhangjinxia@caas.cn (J.Z.); 2 Key Laboratory of Microbial Resources, Ministry of Agriculture, Beijing 100081, China

**Keywords:** *Pleurotus ostreatus*, catalase, expression analysis, development, heat stress

## Abstract

Catalases are ubiquitous hydrogen peroxide-detoxifying enzymes. They participate in fungal growth and development, such as mycelial growth and cellular differentiation, and in protecting fungi from oxidative damage under stressful conditions. To investigate the potential functions of catalases in *Pleurotus ostreatus*, we obtained two catalase genes from a draft genome sequence of *P. ostreatus*, and cloned and characterized them (*Po-cat1* and *Po-cat2*). *Po-cat1* (group II) and *Po-cat2* (group III) encoded putative peptides of 745 and 528 amino acids, respectively. Furthermore, the gene structures were variant between *Po-cat1* and *Po-cat2*. Further research revealed that these two catalase genes have divergent expression patterns during different developmental stages. *Po-cat1*/Po-cat1 was at a barely detectable level in mycelia, accumulated gradually during reproductive growth, and was maximal in separated spores. But no catalase activity of Po-cat1 was detected by native-PAGE during any part of the developmental stages. In contrast, high *Po-cat2*/Po-cat2 expression and Po-cat2 activity found in mycelia were gradually lost during reproductive growth, and at a minimal level in separated spores. In addition, these two genes responded differentially under 32 °C and 40 °C heat stresses. *Po-cat1* was up-regulated under both temperature conditions, while *Po-cat2* was up-regulated at 32 °C but down-regulated at 40 °C. The accumulation of catalase proteins correlated with gene expression. These results indicate that the two divergent catalases in *P. ostreatus* may play different roles during development and under heat stress.

## 1. Introduction

As central components of the enzymatic detoxification pathways, catalases can alleviate oxidative damage by catalyzing hydrogen peroxide (H_2_O_2_) to water and molecular oxygen [1,2]. Catalase is a tetramer metalloenzyme that is composed of four subunits and divided into three gene families, namely monofunctional catalases (typical heme catalases, KatEs), bifunctional catalases (heme catalase-peroxidases, KatGs), and manganese catalases (non-heme catalases, MnCats) [3]. Generally, multiple catalases exist in a single fungus, with each performing specialized functions in development and stress resistance [4,5,6,7]. For instance, *Neurospora crassa* has four catalases: CAT-1 and CAT-3 are two large subunit monofunctional catalases normally expressed in conidia and growing hyphae respectively; CAT-2 is a bifunctional (catalase-peroxidase) member expressed in heat-stressed mycelia; and CAT-4 is a small-subunit monofunctional catalase with unknown function [8,9]. For *Aspergillus nidulans*, CatA is a spore-specific catalase and is not developmentally regulated, CatB is developmentally regulated and is induced by H_2_O_2_, heat shock, and paraquat [10,11]. The catalase family of *Beauveria bassiana* consists of catA (spore-specific), catB (secreted), catP (peroxisomal), catC (cytoplasmic) and catD (secreted peroxidase/catalase) [12]. All of these catalases differ in phylogeny, structure and functional role in resistance toward environmental stresses.

Recently, the mechanisms of fruiting body development have been a popular topic in mushroom studies [13,14,15], and some development-related functional genes and signaling pathways have been identified and characterized, such as nicotinamide adenine dinucleotide phosphate oxidases [16], cytochrome P450 genes (CYPs) [17], superoxide dismutases (SODs) [18], and the cyclic AMP signaling pathway [19]. However, the role of catalase in the development of edible mushrooms has not been reported. Under the condition of horticultural facilities, high temperature is one of the most common environmental factors that can negatively affect the cultivation of mushrooms. Research on thermotolerance mechanism of edible mushrooms has revealed that heat stress can induce apoptosis-like cell death [20], and inhibit mycelial growth and fruiting body formation [21]. Lu et al. [22] and Meng et al. [23] reported elevated catalase activity in *Agaricus bisporus* and *Pleurotus eryngii* var. *tuoliensis* mycelia under heat stress, respectively. However, both studies were based on total catalase activity and did not distinguish specific functions of the separate catalases.

In China, *Pleurotus ostreatus* is mostly cultivated under horticultural facilities, and has the third largest production levels, with a yield of 590.18 million tons in 2015 according to the data of China Edible Fungi Association [24]. Insufficient information exists on mushroom catalases during development and under stress, and hence, further studies are needed to gain a deeper insight. In this study, we cloned and characterized the catalase genes of *P. ostreatus* and analyzed their expression patterns during different developmental stages and under heat stress. 

## 2. Materials and Methods

### 2.1. Strains, Culture Conditions and Genome Sequence Information

The dikaryotic *P. ostreatus* strain CCMSSC00389 (China Center for Mushroom Spawn Standards and Control, Beijing, China) was used in this study and was maintained on potato dextrose agar (PDA) at 4 °C. The corresponding genome and protein sequences are available at DDBJ/EMBL/GenBank under the accession number MAYC00000000 [25,26]. *Escherichia coli* DH5α (Tiangen, Beijing, China) used for plasmid construction was grown in Luria-Bertani broth containing ampicillin (100 μg/mL) or kanamycin (50 μg/mL).

### 2.2. Catalase Identification, Gene Cloning and Sequence Analysis

Catalase genes were obtained from the annotated genome database of *P. ostreatus* strain PC15 [27], and two protein sequences were obtained: 1090819 and 1111887. Then, these two sequences were used to BLAST [28] against the CCMSSC00389 genome database to identify homologs. The nucleotide sequences were used to design primers (*Po-cat1* and *Po-cat2* in Table 1) to amplify the full-length sequences from CCMSSC00389 complementary DNA (cDNA) (details on RNA extraction and processing follow below). The amplified products were purified and cloned into the pGEM-T vector (Promega, Madison, WI, USA) for sequencing. 

The theoretical isoelectric point and molecular weight were computed using the online Compute pI/Mw tool [29]. Transcription factor binding sites were predicted using PROMO [30]. SignalP 4.1 Server [31] was used to predict signal peptides. Conserved domains were analyzed using the online tool [32]. An additional 25 fungal catalase protein sequences were collected from GenBank and JGI. Multiple sequence alignment was performed using MUSCLE [33], and a neighbor-joining (NJ) phylogenetic tree was constructed using MEGA 6.0 [34] with 1000 bootstrap replicates. The gene structure was investigated using Gene Structure Draw Server [35] based on the coding sequences and corresponding genomic sequences.

### 2.3. Fruiting-Body Growth and Sample Collection

The strain CCMSSC00389 was cultured on PDA medium at 28 °C for 7 days. Afterward, a part of the mycelia was collected and the rest was inoculated onto culture compost, which contained 94% cottonseed hull, 5% wheat bran, 1% gypsum and a final water content of 65%. For fruiting body production, the temperature was set at 20 °C (day, 12 h at 500 lux) and at 10 °C (night, 12 h dark), and the room humidity was maintained at 85%. Three samples were collected, including primordia, fruiting bodies and separated spores. Five replicates were collected for each sample, and were frozen in liquid nitrogen immediately after collection.

### 2.4. Heat Stress Treatment

Mycelial growth rate was measured to select suitable heat stress treatment conditions. The determination of growth rate was performed as follows: fresh mycelia were inoculated on PDA medium and cultured at different temperatures (28, 30, 32, 34, 36, 38, and 40 °C) in the dark for 5 days, and then the mycelial diameters were measured. The growth rate was expressed as cm/d. According to the growth rate test and the results of Zhang et al. [36], 32 °C and 40 °C were selected for heat stress treatment. Intracellular H_2_O_2_ content was determined using the Hydrogen Peroxide Assay Kit (Nanjing Jiancheng Bioengineering Institute, Nanjing, China) according to the manufacturer’s instructions. 

For heat stress treatment, the mycelia were first cultured on PDA at 28 °C in the dark for 5 days, and then were transferred to the corresponding temperature for a further 48 h incubation following method of Zhang et al. with cultures at 28 °C used as controls [36]. After treatment, mycelia of eight plates were quickly scraped, mixed, and then quickly frozen in liquid nitrogen and stored at −80 °C for further use. 

### 2.5. Protein Extraction and Catalase Activity Assay

Proteins were extracted using Animal–Plant Total Protein Miniprep Kit (Tiandz, Beijing, China) following the manufacturer’s instructions. Proteins were quantified by measuring absorbance at 595 nm using the BCA Protein Assay Kit (Tiandz) with bovine serum albumin as the standard.

Catalase activity was determined using two different methods, namely, the non-denaturing polyacrylamide gel electrophoresis (native-PAGE) [37] and ultraviolet (UV) spectrophotometry [38]. The native-PAGE was performed following Wang et al. [39], and specific procedures are as follows: equal amounts of total protein (3 μg) were loaded into a 7.5% native polyacrylamide gel for electrophoretic separation, the gel was immersed in 7 mM H_2_O_2_ for 5 min, and then in a 1/1 mixture of freshly prepared 1% potassium hexacyanoferrate (III) and 1% iron (III) chloride hexahydrate. Catalase activity was visualized as a band where H_2_O_2_ was decomposed by catalase. For the UV spectrophotometry assay, catalase activity was measured as the rate of H_2_O_2_ decomposition at 240 nm at 25 °C. A 0.01 decrease in absorbance per second was defined as one unit, and catalase activity was expressed as the number of units per milligram of protein.

### 2.6. Western Blot Analysis

Western blot analysis was performed according to a previous study [40]. Briefly, equal amounts of total protein (20 μg) were loaded into the protein lane and separated in a 12% (*w*/*v*) sodium dodecyl sulfate PAGE gel. After electrophoresis, proteins were transferred onto a polyvinylidene fluoride membrane. Western blot analysis was performed using antibodies against Po-cat1 and Po-cat2, and glyceraldehyde 3-phosphate dehydrogenase (GAPDH, PC15_1090663 (jgi)) was used as control.

### 2.7. RNA Extraction, Reverse Transcription and Quantitative PCR (qPCR)

Total RNA was extracted from 30 mg of −80 °C frozen tissue using the E.Z.N.A. Plant RNA Kit (Omega Bio-Tek, Norcross, GA, USA) following the extraction method for fungal samples, with the addition of DNase I to eliminate genomic DNA. The integrity and concentration of RNA were estimated with an Agilent 2100 bioanalyzer (Agilent Technologies, Palo Alto, CA, USA) using the Total RNA Nano Kit (RNA 6000 Nano LabChip) [41]. The purity of total RNA was measured based on the 260/280 nm absorbance ratio on a Nanodrop 2000 (Thermo Scientific, Wilmington, DC, USA). First strand cDNA was synthesized using the TransScript One-Step genomic DNA Removal and cDNA Synthesis SuperMix Kit (TransGen Biotech, Beijing, China) according to the manufacturer’s instructions.

The KAPA SYBR FAST qPCR Master Mix Kit (Kapa Biosystems, Wilmington, MA, USA) and the ABI 7500 Real-Time PCR amplifier (Applied Biosystems, Foster City, CA, USA) were used for qPCR. All reactions were carried out at a total volume of 20 μL, which contained 2 μL of diluted cDNA, 0.8 μL of primer mix (10 μM), 6.8 μL of nuclease-free water, 0.4 μL ROX Low and 10 μL of SYBR Green mix. All reactions were performed in triplicate. The qPCR amplification procedures were as follows: 95 °C for 3 min, 40 cycles of 95 °C for 3 s, 60 °C for 32 s, and a final extension at 72 °C for 30 s. The GAPDH-encoding gene *gapdh* was used as the reference. Primers were designed using the DNAMAN software v5.2.2 (Lynnon LLC, San Ramon, CA, USA) (Table 1) and were synthesized by Sangon Biotech Co., Ltd. (Shanghai, China). 

### 2.8 Liquid Chromatogram-Tandem Mass Spectrometry Analysis 

Protein bands were cut out directly from the native-PAGE gel and treated with enzyme digestion solution. Afterward, liquid chromatogram-tandem mass spectrometry (LC-MS/MS) was performed to collect and analyze peptide fingerprints. The peptides were searched against the protein sequence of CCMSSC00389 using BLAST [28] to determine protein identity.

### 2.9. Data Analysis

The relative expression of the genes was calculated using the 2^−ΔΔ*C*t^ method [42]. Gene expression levels in the mycelia was used as a reference in the ΔΔ*C*_t_ calculation during different developmental stages, and mycelia cultured at 28 °C was used as a reference in heat stress treatment. The means and standard deviations were calculated from the experiments performed in triplicate. For comparison between the different groups, gene expressions with ≥2-fold changes (up or down) were considered to be different [43]. For other data (catalase activity, mycelial growth rate and intracellular H_2_O_2_ content), parametric one-way analysis of variance (ANOVA) followed by Duncan’s test was used to calculate significant differences among different groups (*p* < 0.05).

## 3. Results

### 3.1. Cloning and Analysis of Catalase Encoding Genes

Two catalase genes were identified in the *P. ostreatus* genome, and were named *Po-cat1* and *Po-cat2*. The cDNA full length sequences of *Po-cat1* and *Po-cat2* were 2238 and 1587 bp, respectively, and DNA sequence analysis revealed eight exons interrupted by seven introns in both genes. The *Po-cat1* and *Po-cat2* sequences were assigned GenBank accession numbers MF491446 and MF491447, respectively. *Po-cat1* encoded a putative 745-amino-acid polypeptide (Po-cat1) of 82.92 kDa with a predicted isoelectric point of 6.02 and *Po-cat2* encoded a putative 528-amino-acid polypeptide (Po-cat2) of 59.72 kDa with a predicted isoelectric point of 6.67. Domain analyses revealed that a heme binding pocket and a tetramer interface in both Po-cat1 and Po-cat2, but an NADPH binding site only in Po-cat2. Both Po-cat1 and Po-cat2 showed no obvious secretory signal sequence. The promoter regions of both *Po-cat1* and *Po-cat2* had binding sites for heat shock factor 1 (Hsf1) which is a stress response transcription factor playing important roles in development and stress resistance [44]. 

**Figure 1:**
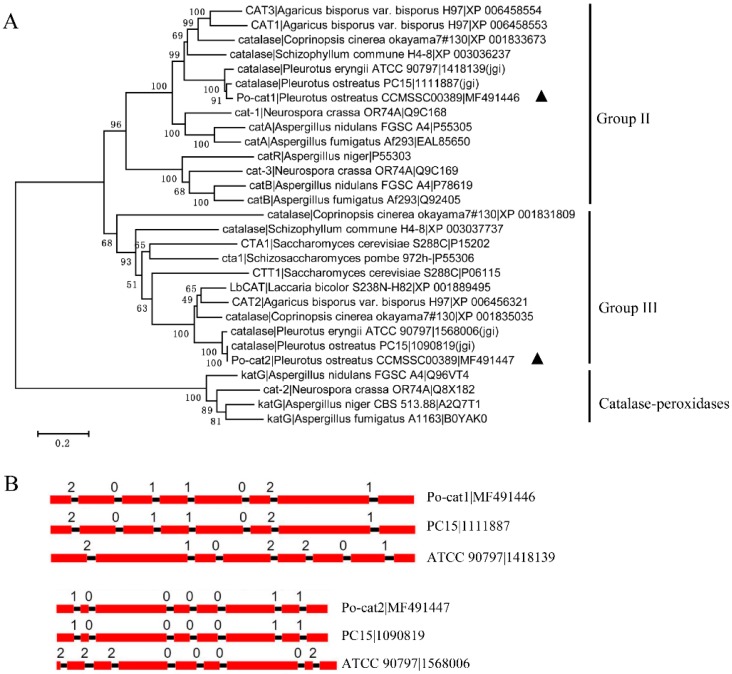
Relationships of fungal catalases and gene structural features. (**A**) A neighbor-joining phylogenetic tree of catalase protein sequences from multiple species; Po-cat1 and Po-cat2 are labeled following with black triangles; (**B**) Gene structures of selected catalase genes, the exons are represented by red rectangles, the black lines connecting two exons represent introns, and the numbers above the line represent the intron phase.

A total of 29 fungal catalase sequences were used for phylogenetic analyses, and these sequences clustered into three clades of group II, group III and catalase-peroxidases (Figure 1A). Po-cat1 and Po-cat2 were monofunctional catalases, and were classified into group II (large subunit enzymes) and group III (small subunit enzymes), respectively. The phylogenetic tree showed that Po-cat1 and Po-cat2 had higher similarities to catalase protein sequences of other mushrooms or fungi than to each other. Po-cat1 had the closest relationship to *P. ostreatus* PC15_1111887 (jgi) and *P. eryngii* ATCC 90797_1418139 (jgi), while Po-cat2 had the closest relationship to *P. ostreatus* PC15_1090819 (jgi) and *P. eryngii* ATCC 90797_1568006 (jgi). 

*Po-cat1* had an identical gene structure with the gene encoding PC15_1111887, and *Po-cat2* had an identical gene structure with the gene encoding PC15_1090819 (Figure 1B). However, *P. ostreatus* and *P. eryngii* had different gene structures even though their catalase protein sequences were highly similar. Phylogenetic and gene structure analyses provided evidence that *Po-cat1* and *Po-cat2* have high identity to catalases of other fungal species, but are not genetically closely related to one another.

### 3.2. Expression Patterns of Catalases during Different Developmental Stages of P. ostreatus

**Figure 2:**
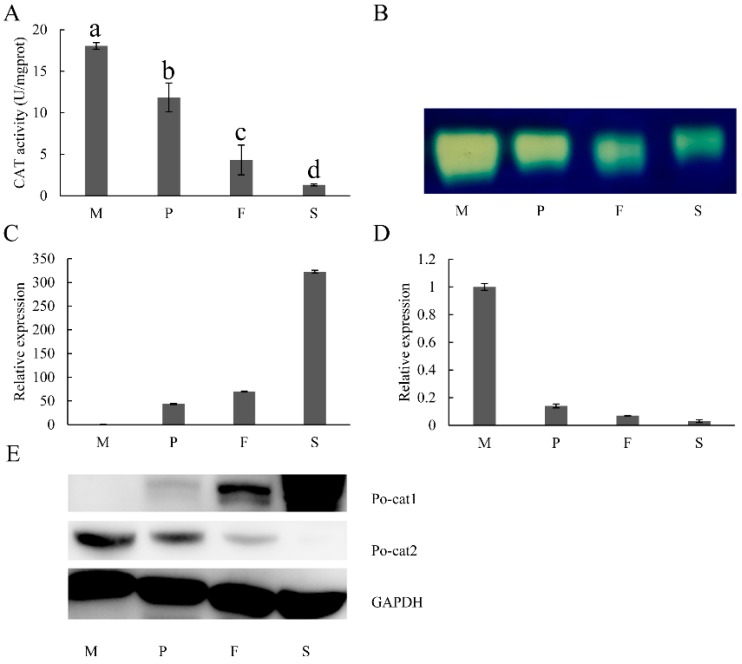
Expression patterns of catalases during different developmental stages of *P. ostreatus*. (**A**) Total catalase activities at different growth stages; (**B**) Po-cat2 catalase activity; (**C**) Differential expression of *Po-cat1*; (**D**) Differential expression of *Po-cat2*; (**E**) Protein expression of Po-cat1 and Po-cat2. M = mycelia; P = primordia; F = fruiting bodies; S = spores. The gene expression levels are presented relative to that in mycelia. Mean values and standard deviations of three biological replicates are shown. The error bars with different letters over the columns denote significant differences (*p* < 0.05, *n* = 3).

To investigate the expression patterns of catalases in *P. ostreatus* during development, the detection of mRNA level, protein accumulation and catalase activity were performed. As revealed by UV spectrophotometry and native-PAGE, the highest catalase activity was observed in mycelia, followed by primordia, then fruiting bodies, and reached the minimal level in the separated spores (Figure 2A,B). Only one catalytic band was detected by native-PAGE (Figure 2B), while there are two catalase genes found in the genome. The results of LC-MS/MS verified that these observed bands were polypeptides of Po-cat2 (the information on detected Po-cat2 polypeptides in mycelia can be found in supplementary Figure S1). This suggests that, of the two catalases, only Po-cat2 plays a catalytic function in decomposing H_2_O_2_ during any part of the life cycle of *P. ostreatus*.

The analyses of quantitative real-time PCR revealed that the mRNA levels of *Po-cat1* were up-regulated significantly and continuously during developmental stages, being 44-fold (primordia), 70-fold (fruiting bodies), and 323-fold (spores) higher than that of the mycelia (Figure 2C). Expression of Po-cat1 protein illustrated by Western blot showed the same trend with mRNA of *Po-cat1*, which was hardly detectable in mycelia and maximal in spores (Figure 2E). The extremely high mRNA and protein expression levels of *Po-cat1* were seemingly inconsistent with the lack of observed catalase activity (Figure 2B). The mRNA level of *Po-cat2* was down-regulated, being 0.14-fold (primordia), 0.07-fold (fruiting bodies), and 0.03-fold (spores) of that of the mycelia (Figure 2D). Expression of the Po-cat2 protein showed a similar pattern, being highest in mycelia and lowest in spores. For *Po-cat2*, the consistent expression trends of mRNA, protein and enzyme activity suggest that Po-cat2 was possibly regulated at the transcriptional level.

### 3.3. Selection of Heat Stress Conditions

**Figure 3:**
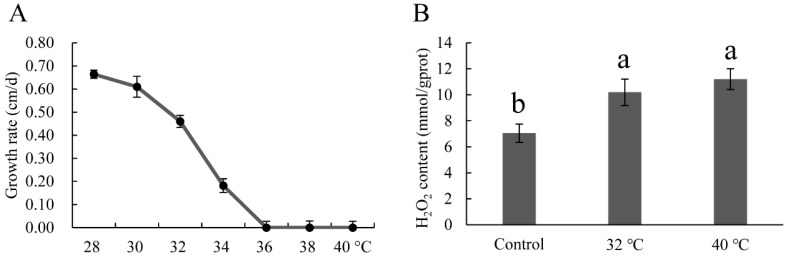
Growth rate (**A**) and H_2_O_2_ contents (**B**) of mycelia at different growth temperatures. Mean values and standard deviations of three biological replicates are shown. The error bars with different letters over the columns denote significant differences (*p* < 0.05, *n* = 3).

To select suitable heat stress conditions, a mycelial growth rate test was performed. As shown in Figure 3A, mycelial growth rate decreased gradually with the increase in temperature, and reached zero at 36 °C. According to the results of Zhang et al., 40 °C is the optimal heat stress temperature for *P. ostreatus* [36]. The two temperatures, 32 °C at which the mycelial growth was affected slightly, and 40 °C at which the mycelial growth was completely inhibited were used to conduct the heat stress treatment. Hydrogen peroxide, which is an indicator of oxidative damage, was measured. In this study, the H_2_O_2_ contents under 32 °C and 40 °C were higher than that of the control (28 °C), indicating that oxidative damage occurred under both heat stress conditions.

### 3.4. Expression Patterns of Catalases under Heat Stress

**Figure 4:**
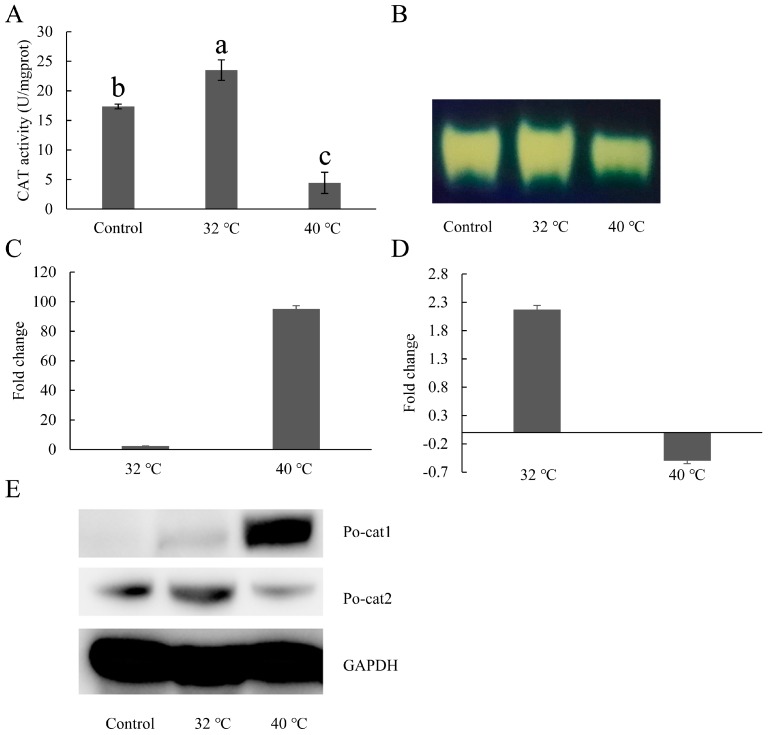
Expression patterns of catalases under heat stress. (**A**) Total catalase activities after 32 °C and 40 °C heat stress; (**B**) Po-cat2 catalase activity; (**C**) Expression patterns of *Po-cat1* under heat stress; (**D**) Expression patterns of *Po-cat2* under heat stress; (**E**) Protein expression patterns of Po-cat1 and Po-cat2 under heat stress. The gene expression levels are presented relative to mycelia cultured at 28 °C. Mean values and standard deviations of three biological replicates are shown. The error bars with different letters over the columns denote significant differences (*p* < 0.05, *n* = 3).

To investigate the expression patterns of catalases in *P. ostreatus* under heat stress, mRNA level, protein accumulation and catalase activity were assessed. Catalase activity (Po-cat2 catalase activity) increased under 32 °C, but decreased under 40 °C (Figure 4A,B). The transcript levels of *Po-cat1* and *Po-cat2* in the 32 °C-treated mycelia increased by 2.3-fold and 2.1-fold compared to that of the 28 °C control, respectively (Figure 4C). The transcript levels of *Po-cat1* increased by 95-fold while *Po-cat2* decreased by 0.5-fold under 40 °C heat stress (Figure 4D). Protein accumulation of Po-cat1 and Po-cat2 showed the same expression profiles as mRNA levels. The above results suggest that both the *Po-cat1* and *Po-cat2* are involved in heat resistance but in different roles.

## 4. Discussion

Catalases are ubiquitous in nature and are found in all kingdoms of life. The roles of catalase homologs are of great research interest because their specific functions have not been completely elucidated. Twenty nine catalase sequences were used for phylogenetic analysis of which fifteen sequences were from mushroom species. As shown in Figure 1A, mushroom catalases exhibited different levels of diversity. Our sequence analysis showed significant differences between *Po-cat1* and *Po-cat2*. These two sequences were not found to be phylogenetically closely related to each other and have been classified into different gene families. In the phylogenetic tree, they appear as paralogs, indicating a more ancient origin rather than recent gene duplication.

*Po-cat1*/Po-cat1 and *Po-cat2*/Po-cat2 showed opposite expression trends during different stages of development. *Po-cat1/*Po-cat1 was barely detectable in growing mycelia, highly induced during reproductive growth, and maximal in spores, and thus, *Po-cat1* is considered as a spore-specific catalase which might participate in spore-related processes. Our clustering analysis supports this view, as spore-specific catalases of other fungi clustered into the same branch with Po-cat1, such as CatA of *Aspergillus fumigatus* (EAL85650) [12,45], CAT-1 of *N. crassa* (Q9C168) [9], and CatA of *A. nidulans* (P55305) [10]. The high accumulation of *Po-cat1*/Po-cat1 showed no detectable catalase activity in the tested conditions. This phenomenon is quite similar to the catalases of *B*. *bassiana* and *Cryptococcus neoformans*. In *B*. *bassiana*, the five catalases had different levels of transcripts, but it seems that only catB and catP could be normally active, since only two catalase-active bands rather than the expected five bands were visible on the native-PAGE [12]. *C. neoformans* possesses four catalases (*CAT1*, *CAT2*, *CAT3*, and *CAT4*) and transcripts for each of them can be detected by qPCR, but only Cat1 was a functionally active catalase under both normal and stressful conditions [46]. In this study, we speculate that no active Po-cat1 complex (tetramer) is formed, or that it engages in other catalytic functions, and this needs further validation. Po-cat2 was the predominant catalytic catalase enzyme during the entire life cycle, accumulating maximally in mycelia, gradually decreasing during reproductive growth, and minimal in spores. This phenomenon is different in *A. nidulans* [11], *Paracoccidioides brasiliensis* [4], and *N. crassa* [47]. All these three species have high catalase activity both in spores and during vegetative growth, which are represented by different catalases. The low activity in spores of *P. ostreatus* may suggest that huge differences in catalase may exist between mushrooms and other fungi. In addition, alternative H_2_O_2_ detoxification pathways, such as glutathione peroxidase and thioredoxin reductase systems, may function in spores of *P. ostreatus* [48].

Several researchers have reported the induction of catalases during heat stress for multiple species, such as *Saccharomyces cerevisiae* [49], *Rhodotorula mucilaginosa* [50], *A. nidulans* [10], *Penicillium marneffei* [51], and *P. brasiliensis* [4]. In this study, the two catalase genes responded differentially under 32 °C and 40 °C heat stresses. *Po-cat1*/Po-cat1 was significantly induced during heat stress, especially under 40 °C, but its high accumulation was not associated with any catalase activity. *Po-cat2*/Po-cat2 was induced under 32 °C heat stress, and the increased Po-cat2 activity could enhance the resistance of mycelia to adverse external environmental conditions. But Po-cat2 activity decreased under 40 °C heat stress. This is interesting, because one would expect that under this more stressful condition, the mycelia would synthesize more enzymes required for the decomposition of H_2_O_2_. In fact, decreased catalase activity under abiotic stresses has been found in many plants and fungi, such as *Pisum sativum* L. [52], *Brassica napus* [53], *Triticum aestivum* L. [54], and some strains of *Candida* species [55]. One possible reason for the decrease in Po-cat2 activity may be the inhibition of overall protein synthesis under stressful conditions, as has been previously shown [56]. Another possible reason may be that alternative H_2_O_2_ detoxification pathways function in this situation [48].

## 5. Conclusions

In this study, we cloned and characterized the *P. ostreatus* genes, *Po-cat1* and *Po-cat2*, which show sequence similarity to catalase genes. We analyzed their expression patterns during several growth stages and under heat stress. These two genes are diverse in structure and have diametrically opposed expression patterns during different developmental stages, and they also respond differentially under 32 °C and 40 °C heat stress conditions. Only *Po-cat2* plays a catalytic function in decomposing H_2_O_2_ during any part of the life cycle of *P. ostreatus*, while the function of *Po-cat1* is unknown. Further studies are required to reveal the specific function of *Po-cat1* which has high expression during some stages but no detectable catalase activity. These results indicate that the two genes may play different roles during development and under heat stress. This study provides a greater understanding of the biological functions of catalases in edible mushrooms. This study also provides a reference for the elucidation of the mechanisms involved in mushroom development and stress resistance.

## Figures and Tables

**Table 1:** Primers used in complementary DNA full length sequence amplification and quantitative PCR (qPCR).

Name	Forward Sequence (5′–3′)	Reverse Sequence (5′–3′)	Product Size (bp)
*Po-cat1*	ATGTCGTCCATCACAGCTG	TCAATACGCAATCCTCGC	2238
*Po-cat2*	ATGCCCACTCAAGAAGTC	TCAGTGGGCGGTGGACTT	1587
*gapdh*	GTGTTAACCTCGAGACTTACG	TGGTGGCGTGGATTGTGCTC	144
*Po-cat1_1*	TGTGCATTGGTTGAGAGAGG	TACGACGCTACAACTTCCG	147
*Po-cat2_1*	CGGACTTTCTTGCCCACAG	GACTTGCTCGCCCATTTCG	149

## Supplementary Materials

The following are available online at www.mdpi.com/2073-4425/8/11/335/s1. Figure S1: The information on detected Po-cat2 polypeptides in mycelia by LC-MS/MS analysis.
