# Supplementary material for: Differential Expression Patterns of Pleurotus ostreatus Catalase Genes during Developmental Stages and under Heat Stress
Source: Genes (Basel). 2017 Nov 21;8(11):335. doi: 10.3390/genes8110335 (PMC5704248; doi:10.3390/genes8110335)
Supplement: Supplementary file 1 [file genes-08-00335-s001.docx]

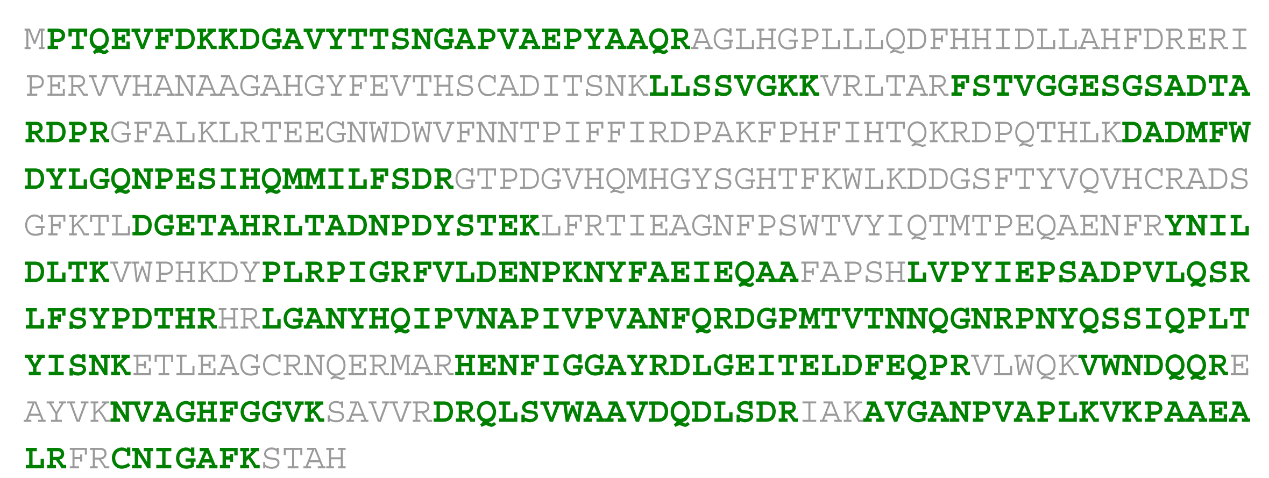


**Figure S1.** The information on detected Po-cat2 polypeptides in mycelia by LC-MS/MS analysis. The gray capitals represent putative amino acid sequences of Po-cat2, the green capitals represent sequences detected by LC-MS/MS of Po-cat2.
